# Early recognition of familiar word-forms as a function of production skills

**DOI:** 10.3389/fpsyg.2022.947245

**Published:** 2022-09-16

**Authors:** Irene Lorenzini, Thierry Nazzi

**Affiliations:** Université Paris Cité, CNRS, Integrative Neuroscience and Cognition Center, Paris, France

**Keywords:** early word-form recognition, early word-form processing, perception-production link, infant speech perception, infant speech production

## Abstract

Growing evidence shows that early speech processing relies on information extracted from speech production. In particular, production skills are linked to word-form processing, as more advanced producers prefer listening to pseudowords containing consonants they do not yet produce. However, it is unclear whether production affects word-form encoding (the translation of perceived phonological information into a memory trace) and/or recognition (the automatic retrieval of a stored item). Distinguishing recognition from encoding makes it possible to explore whether sensorimotor information is stored in long-term phonological representations (and thus, retrieved during recognition) or is processed when encoding a new item, but not necessarily when retrieving a stored item. In this study, we asked whether speech-related sensorimotor information is retained in long-term representations of word-forms. To this aim, we tested the effect of production on the recognition of ecologically learned, real familiar word-forms. Testing these items allowed to assess the effect of sensorimotor information in a context in which encoding did not happen during testing itself. Two groups of French-learning monolinguals (11- and 14-month-olds) participated in the study. Using the Headturn Preference Procedure, each group heard two lists, each containing 10 familiar word-forms composed of either early-learned consonants (commonly produced by French-learners at these ages) or late-learned consonants (more rarely produced at these ages). We hypothesized differences in listening preferences as a function of word-list and/or production skills. At both 11 and 14 months, babbling skills modulated orientation times to the word-lists containing late-learned consonants. This specific effect establishes that speech production impacts familiar word-form recognition by 11 months, suggesting that sensorimotor information is retained in long-term word-form representations and accessed during word-form processing.

## Introduction

The relationship between speech perception and production in early phonological development is receiving growing attention. Perception/production links have been reported, between 6 and 18 months of age, for a wide range of experimental tasks, comprising audiovisual matching (Streri et al., 2016; Altvater-Mackensen et al., 2016); unfamiliar word-form processing (DePaolis et al., 2011, 2013; Majorano et al., 2014); speech segmentation (Hoareau et al., 2019); familiar words mispronunciation detection (Altvater-Mackensen et al., 2014); word learning (Altvater-Mackensen and Fikkert, 2010; Majorano et al., 2019) and categorical perception (Vilain et al., 2019). These studies show a boosting effect of growing production skills on perceptual processing and their shared interpretation is that early practice of speech sounds opens up a novel source of phonological knowledge: sensorimotor knowledge [i.e., phonological knowledge derived from speech-related motor and proprioceptive practice, cf. Guellaï et al. (2014)]. Following the setting of this perception/production coupling, the representation of “own” speech sounds (sounds that have been practiced by the infant) would become stronger (stronger memory trace, easier access) compared with the representation of “non-own” speech sounds, only experienced through the input.

In this perspective, babbling has been proposed as the basic mechanism underlying the setting of the perception/production coupling, a perceptuomotor activity allowing the perceptuo-cognitive connection between one’s speech actions and the proprioceptive and acoustic percepts generated by this action (e.g., Vihman et al., 2009). Neurofunctional evidence supports these hypotheses. Imada et al. (2006) investigated the respective activation to CV syllables of the superior temporal cortex (locus of auditory analysis) and inferior frontal regions (involved in speech motor analysis) from 0 to 12 months. They found that, from 6 months onward, activity in the superior temporal regions was progressively mirrored by activity in the inferior frontal regions. This trend was interpreted as evidence of the setting of a perceptuo-motor link, tentatively related to the onset of babbling-like movements.

Going beyond purely linguistic tasks, Keren-Portnoy et al. (2010) investigated the relationship between the development of speech production skills and of interrelated memory functions (phonological working memory). The authors found a significant correlation between speech production skills (length of experience with babbling) and phonological working memory as measured at 24 months in a word repetition task. They also found better retention, in the repetition task, of word-forms embedding consonants that were part of the participants’ babbling repertoires.

This topic has been recently rediscussed in a very comprehensive review by Vihman (2022), arguing that speech sound production and its interaction with the perceptual system might be fundamental for the development of phonological memory.

All in all, the interaction between perception and production during infancy has been linked to the development of a wide set of linguistic processing mechanisms as well as to the development of memory capacities. In this context, the onset of production has often been taken as the measure of reference, the point of departure of the perception/production link. However, going beyond this principle, a parallel line of investigation has shown that perception/production effects can also be observed before the onset of speech production, as early as at 3 months of age. At this early stage, fundamental perceptual capacities such as phoneme discrimination and audiovisual matching can be modulated by the administration of teething toys, which enhance or inhibit perception depending on whether they mirror or block the movements underlying the production of the heard sounds (Yeung and Werker, 2013; Bruderer et al., 2015; Choi et al., 2019, 2021). Importantly, neurofunctional evidence has confirmed that the interference obtained is perceptuo-motor rather than due to a general disturbance effect (Choi et al., 2021). Valuably amending previous conclusions, and showing that action/perception mechanisms in speech perception might be active from very early in life, these studies promote a debate that echoes investigations targeting the ontogeny of general action/perception mechanisms in the human brain. For instance, Del Giudice et al. (2009) proposed that mirror neurons might result from Hebbian learning allowed by the possibility to perceive one’s own action in infancy, while at the same time underlining that infants’ perceptual-motor system might be particularly efficient in providing input for Hebbian learning.

In summary, the perception/production coupling in speech development is precocious (preceding, in some form, experience with speech production) and consistent (spanning different developmental periods). However, more research is needed to clarify both the temporal timeline and the functional dynamics of this perception/action mechanism: is it active from birth? How is it modulated by the onset of speech production? In which ways does it influence phonological development? Research and debate on these points are just at their beginnings (Choi et al., 2019; Vilain et al., 2019).

### The perception/production link in early word-form processing

The specific object of this study, the speech perception/production link in early word-form processing, has been addressed in foundational studies in the field. Capitalizing on extensive first-hand analyses of production, DePaolis et al. (2011) tested listening preferences around 10 months of age for pseudowords containing consonants that each participant: either regularly produced in babbling (“own-consonants”); or did not produce, but are common at the age tested (i.e., are within their reach in terms of articulatory development); or did not produce, and are generally not produced at the age tested (exceeding current articulatory development). The study revealed a significant preference, in more advanced babblers (infants who mastered at least two supraglottal consonants), for non-mastered, yet producible consonants. These results were replicated and extended in a longitudinal study by Majorano et al. (2014), who additionally reported longer listening in less advanced babblers for pseudowords containing own-consonants. No preference for any of the given consonant categories was found in pre-babbling infants.

Such patterns have been interpreted in agreement with the “Articulatory Filter Hypothesis.” Originally formulated by Vihman (1993), this perspective made the hypothesis that the perception/production coupling of speech sounds, as obtained through babbling practice, modifies infants’ attention to the speech signal. In other words, when an infant starts stably producing a new consonant, the representation of such a consonant integrates sensorimotor information and becomes richer, thus particularly salient in the speech stream. The investigations by DePaolis and colleagues and Majorano and colleagues were designed to test such hypothesis, for which they both provided empirical support. Beyond this, the finding of a preference for non-mastered consonants in more advanced producers by DePaolis et al. (2011), followed by the finding of a preference for own-consonants in less advanced producers by Majorano et al. (2014), were unexpected. This pattern was taken to show that well-practiced sounds become «overly familiar» stimuli, that start being processed with lesser cognitive effort than non-own consonants and trigger less attention (DePaolis et al., 2011, p. 598).

To sum up, the Articulatory Filter Hypothesis posits a form of representational coupling of phonological knowledge derived on the one side from perception and on the other side from production, which is hypothesized to support encoding and recognition of word-forms, as well as word-object learning. Here, we followed up on these previous results, and explored retention of sensorimotor information in long-term word-form representations.

## The present study: Distinguishing encoding from recognition to test long-term word-form representations

*Encoding* can be defined as the process of translating information into a format that can be stored in the memory system; *recognition* as the automatic retrieval of a stored item (a word-form, in our case), triggered by the occurrence of an instance of such item (Melton, 1963; Atkinson and Shiffrin, 1968; Gillund and Shiffrin, 1984).

Previous studies have targeted the processing of new word-forms (e.g., DePaolis et al., 2011; Majorano et al., 2014). Thus, crucially, the tasks used entailed both an encoding and a recognition phase, and the perception/production effect could come from the former, the latter or both. The present study aimed at clarifying this issue: by using real familiar words, previously learned by participants in their daily environment, we excluded encoding from the experimental task and assessed recognition of word-forms stored in long-term memory.

Distinguishing encoding from recognition has relevant theoretical implications. In fact, showing that sensorimotor information is used for word-form encoding reveals that this information is part of what is processed when learning a new word. However, this does not necessarily imply that sensorimotor information is retained as part of the (multisensory) word-form representation, which is instead suggested if the effect is still obtained testing recognition. Two investigations have addressed this issue so far. Majorano et al. (2019) tested word learning at 11 months of age with an ecological learning procedure followed by a preferential looking task. The target items were composed of either Early-Learned Consonants (ELC) or Late-Learned Consonants (LLC), respectively being or not being part of typical babbling repertoires. The authors obtained evidence of word retention limited to the participants showing more advanced production and to ELC-words, thus signaling a specific effect of speech-related sensorimotor learning on long-term word-form storage. However, evidence of learning was overall weak: more *vs.* less advanced babblers preferred looking at the visual referent associated to ELC-words when it was contrasted with the referent associated to LLC-words, but, when the ELC referent was contrasted with a foil image, the whole group preferred looking at the foil.

In another vein, Laing and Bergelson (2020) analyzed babbling samples recorded from 10-to-11-month-olds and assessed whether or not the consonants uttered matched the name of the objects that the infants were attending to during the recordings, which implies the retrieval of a previously stored phonological form. The results revealed that infants with more advanced production skills were more likely to produce consonants mirroring the name of the contextual object. However, the authors failed to find strong evidence of a specific perception/production relationship (i.e., of babbling participants producing more object-matching consonants when the object’s name contained consonants belonging to their inventories).

With this study, we aimed at building on previous findings, further investigating the issue of the retention of sensorimotor information in long-term word-form representations. To these goals, we measured orientation times to familiar word-forms in French-learning infants displaying more *vs.* less advanced production skills, using the Headturn Preference Procedure without a familiarization phase. Differently from previous studies, we did not select our stimuli to mirror participants’ individual production patterns. We relied on evidence that French-learning infants of the age tested produce a majority of early-learned consonants (plosives and nasals, ELCs) and a minority of late-learned consonants [fricatives and liquids, LLCs; classification and terminology being taken from Majorano et al. (2019)]. In particular, De Boysson-Bardies and Vihman (1991) conducted a longitudinal study on babbling patterns in French learners based on transcription of home-recorded samples. The authors specified consonant count for the age range between 10 and 15 months of age, corresponding to the ages targeted in the present research. They reported no significant change in the relative production of ELC and LLC during this time period and, precisely, a ratio of 80% ELCs to 20% LLCs (Table 3, p. 303 of the original paper). Levitt and Utman (1992) presented a case study whose results agree with the tendencies identified by de Boysson-Bardies & Vihman (although note that the relative percentage of LLCs reported here appears slightly lower than those reported by de Boysson-Bardies & Vihman, which is related to the fact that Levitt & Utman also counted glides, while de Boysson-Bardies & Vihman did not).

Similar patterns are also observed cross-linguistically and beyond individual variation, due to the progressive attainment of neuromuscular control on the articulators (Green et al., 2000; McLeod and Crowe, 2018). On these bases, we contrasted words containing ELCs with words containing LLCs assuming that, at the group level, participants would master ELC production and be at the early stages of LLC production (production skills were then verified on the day of testing). While this methodological choice prevented us from obtaining individual-child results, it allowed us to use a wider variety of speech sounds and to test more homogeneous age groups than in previous studies (DePaolis et al., 2011; Majorano et al., 2014). Such studies have shown a perception/production relationship rooted in individual production patterns. Here, we proceeded in a different way, basing our stimulus selection on principles that are valid beyond individual variation. Note that Altvater-Mackensen et al. (2014) and Majorano et al. (2019) used analogous distinctions.

In a first experiment, we tested a group of 11-month-olds, as word-form recognition has been shown to emerge at that age (e.g., Hallé and de Boysson-Bardies, 1994; Vihman et al., 2004; Swingley, 2005; Poltrock and Nazzi, 2015; DePaolis et al., 2016; Vihman and Majorano, 2017). In Experiment 2, we then targeted a group of 14-month-olds. We chose this second age range because, based on previous evidence on babbling in French-learning 10-to-15-month-olds (De Boysson-Bardies and Vihman, 1991), we expected more advanced consonant production at this stage. Following the Articulatory Filter Hypothesis, wider experience with speech sound production should entail stronger perception/production effects, due to a broadening of the perception/production coupling to several sounds. At both ages, our hypothesis of a role of production in word-form recognition predicted differences in listening preferences as a function of word type (containing ELCs vs. LLCs) and, following DePaolis et al. (2011) and Majorano et al. (2014), of production skills. Specifically, we expected a preference for word-lists containing LLCs, which would be modulated by production level, being possibly stronger, or present only, in more advanced producers.

## Experiment 1

### Materials and methods

#### Participants

Thirty-two full-term healthy infants were included in the study (21 females, 11 males; mean age: 11 months, 12 days; range: 11 months–11 months, 29 days). All participants came from monolingual French-speaking homes and the parents reported no familial history of speech, language or hearing disorders. Six additional infants were tested but not included due to experimenter’s error (1) or fussiness (5). All parents gave informed consent prior to testing.

#### Production assessment: Parental questionnaire

For each infant, the type and quantity of consonants produced were estimated through a parental questionnaire. Specifically, we used the same questionnaire as Gonzalez-Gomez and Nazzi (2012) and Hoareau et al. (2019), originally adapted from Stoel-Gammon (1989). While, as compared with direct observation, this kind of questionnaire provides an indirect measure of infant production, previous studies have reported that such questionnaires can be reliable. For example, Ramsdell et al. (2012) reported reliability of a similar parental babbling questionnaire, administered to parents of infants aged from 10 to 12 months (in which, specifically, parents were asked to report the sounds produced by their infant in that period). Furthermore, use of the present questionnaire or of very similar versions has previously allowed to detect differences in production skills between groups of full-term and preterm infants at 10 months (Gonzalez-Gomez and Nazzi, 2012), a link between speech production and word-form segmentation abilities at 8 months (Hoareau et al., 2019), and differences in audiovisual matching before and after babbling onset at 9 months (Vilain et al., 2019).

The questionnaire was administered as a checklist in which, for each French consonant, parents had to choose whether their infant produced the consonant: (a) on a regular basis and with a stable and correct phonetic form; (b) sporadically and/or with variable phonetic forms; (c) did not yet produce the consonant. Only the consonants falling in category (a) were retained for analyses. We also asked whether infants produced reduplicated and variegated babbling. This was an exclusion criterion, as severe limitation of babbling patterns at this age might signal atypical production development (e.g., Whitehurst et al., 1991; Oller et al., 1999). No participant had to be excluded on this basis. The questionnaire was filled on the day of testing after the HPP procedure or, if this was not possible, within a week after testing. The questionnaire was administered by the first author in exactly the same way to all parents. Parents did not receive explicit instructions on how to formulate their judgments and none of the families displayed difficulties answering the questions.

#### Headturn preference procedure

##### Stimuli

Twenty familiar words were chosen from the French version of the Communicative Developmental Inventories (CDI; Kern, 2003): 10 words exclusively containing Early-Learned Consonants (ELCs, that emerge early in speech development and are produced through relatively simple articulation patterns: plosives and nasals) and 10 words exclusively containing Late-Learned Consonants (LLCs, entailing more complex articulatory dynamics and, thus, typically acquired later in development: fricatives and the lateral/l/). Vowel context was varied in an effort to balance the different types of vowels (height, openness, rounded/unrounded, nasalization, presence of diphthongs). Adult word frequency, syllabic length and the frequency of the initial, internal and final diphones were controlled between lists based on the Lexique corpus (New et al., 2004) and the Diphones corpus (New and Spinelli, 2013).

Familiar words were identified in the CDI following the familiarity criteria in Vihman et al. (2004) and Poltrock and Nazzi (2015). According to parental reports, the selected items were comprehended on average by 27% (range: 13–58%) of French-learning infants by 11 months of age (S. Kern: personal communication on MacArthur Bates specifying CDI scores in 55 11-month-old French-learners and 50 14-month-old French-learners). The familiarity of the words was also verified for each participant on the testing day. Concerning this point, because we were interested in testing the word-form level [which is phonological in nature and does not need meaning to be included in the representation, Werker and Curtin (2005)], parents were asked to rate how often their infants *heard* (and not how often their infants *comprehended*) the words tested. The possible answers were: 0 = Never; 1 = rarely: once per week; 2 = one time per day; 3 = several times per day. The mean response was 1.9 (SD = 0.45), similarly to Swingley (2005) and Poltrock and Nazzi (2015). Twenty-five infants were familiar with all the words. Of the seven infants who were unfamiliar with one or more of the words, parents chose answers 0 or 1 for a mean number of 4.5 words out of 20. Additionally, we asked families whether the infants were able to produce one or more of the test-words. None of the infants was reported to produce any of the words.

The stimuli were recorded in a sound-proofed booth in Infant Directed Speech by a French-native female speaker using an Audio-Technica ATR-20 microphone. Three tokens of each item were selected and six pseudo-randomized lists were constructed for each condition: six lists of familiar words containing ELCs and six lists of familiar words containing LLCs. In each list, each of the 10 words was presented twice, so that the list contained 20 tokens in total. The order of all items in the lists (including initial items) was pseudo-randomized, ensuring that each word was well-distributed within and across lists. All lists lasted 22.30 s, with an interstimulus interval varying between 550 and 630 ms. The stimuli were controlled for duration, intensity and fundamental frequency (stimuli; means, standard deviations and significance levels are given in Tables 1, 2).

**TABLE 1:** Test stimuli used in experiment 1: familiar words containing early vs. late-learned consonants (ELC; LLC).

Familiar word ELC	Phonetic transcription	Syllabic length	English translation	% French-learning infants familiar with the word	Mean familiarity index (scale: 1–3)	Word frequency (Lexique FreqLemFilms2)	Mean diphone frequency
pain	pɛ̃	1	*bread*	58	1.8	67.58	368
nez	ne	1	*nose*	20	2.5	75.18	NA
doigt	dwa	1	*finger*	15	1.8	85.96	877.50
pied	pje	1	*foot*	36	1.8	214.08	1586.5
pomme	pɔm	1	*apple*	13	2	42.35	741
tête	tεt	1	*head*	18	3	475.87	1746
cube	kyb	1	*cube*	16	1.3	2.81	566
gâteau	gato	2	*cake*	56	1.5	55.19	863
body	bodi	2	*body*	18	1.5	*NA*	1077
banane	banan	2	*banana*	13	2	11.4	1199

**Familiar word LLC**	**Phonetic transcription**	**Syllabic length**	**English translation**	**% French-learning infants familiar with the word**	**Mean familiarity index (scale: 1-3)**	**Word frequency (Lexique FreqLemFilms2)**	**Mean diphone frequency**

chat	∫ a	1	*cat*	51	3	93	850.00
lit	li	1	*bed*	40	2	184.27	2843.00
lait	le	1	*milk*	29	2	59.62	1226.00
verre	vεR	1	*Glass*	13	1.5	176.57	2382
soeur	sæᴚ	1	*sister*	22	1.5	184.99	925
vache	va∫	1	*cow*	20	1.6	47.71	1891
chaise	∫εz	1	*chair*	22	1.5	40.02	978
avion	avjõ	2	*airplane*	15	1.8	128.35	1407
chausson	∫ osɔ̃	2	*slipper*	13	2	3.5	390
chaussure	∫ osyR	2	*shoe*	51	2	73.58	1023
***T*** **-tests (two-sided) ELC vs. LLC words:**				*t*_(18)_ = −0.18; *p* = 0.86	*t*_(18)_ = 0.14; *p* = 0.89	*t*_(18)_ = 0.28; *p* = 0.78	*t*_(18)_ = −1.37; *p* = 0.19

**TABLE 2:** Experiment 1: acoustic characteristics of the stimuli.

	Condition		*t*-value and significance level (two-sided)
	Early-learned C	Late-learned C	
Duration (ms)	570 (SD = 70)	600 (SD = 90)	*t*_(18)_ = −0.40; *p* = 0.69
Amplitude (dB)	74.25 (5)	74.9 (4)	*t*_(18)_ = −0.07; *p* = 0.95
F0 mean (Hz)	267.77 (13.4)	273 (14)	*t*_(18)_ = −0.16; *p* = 0.88
F0 min (Hz)	203.63 (26)	220.9 (32)	*t*_(18)_ = −0.67; *p* = 0.51
F0 max (Hz)	331.8 (16.5)	322.6 (24)	*t*_(18)_ = 0.21; *p* = 0.83

**TABLE 3:** Experiment 1: quantity and type of consonant produced (parental questionnaires. 11-month-olds).

Age (mos.days)	Tot. C produced	Babbling inventory
11.2	3	b k g
11	3	p b m
11.16	4	p b t m
11.23	4	b t d m
11.21	4	p b k m
11.20	4	p b t m
11.4	4	t d m n
11.1	4	t d m n
11	4	t d g m
11.16	5	p d g m n
11.2	5	p b t d m
11.11	5	p b t k m
11.3	5	p b d m l
11.5	5	p b t k g
11.18	5	p b d g l
11.29	5	p b d g l
11.17	5	p b t d m
11.16	5	p t d k l
11.23	5	p t d m n
11.18	5	p b t d m
11.26	6	p b t d g m
11.23	6	p b t d k m
11	6	p b d k g m
11.7	6	p b t d m l
11.10	6	p b t d m n
11.8	6	p b t k g m
11.22	7	p b t d g m n
11.3	7	p b t d k m n
11.9	8	p b t d k g m n
11.9	8	p b t d k g m n
11.10	8	p b t k g m n l
11.10	8	p b t d k g m l

### Procedure and apparatus

The experiment took place in a sound-attenuated booth containing a three-sided test booth made by pegboard panels. The test booth had two red lights and a loudspeaker (SONY xs−F1722) on each side and a green light at the center. All lights were at eye level for the participants. A hidden camera was accommodated in a 5 cm hole below the center light. A PC terminal (Dell Optiplex computer), an audio amplifier (Marantz PM4000), a TV screen connected to the camera, and a three-button response box were located outside the booth. The experimenter monitored the participant’s behavior on the TV screen and pressed the buttons of the response box according to the direction of the infant’s headturns, thus starting and stopping the flashing of the lights and the presentation of the sounds. Orientation times were measured and recorded on-line. The experimenter and the caregiver wore earplugs and listened to music over tight−fitting headphones, preventing them from hearing the stimuli.

We used the Headturn Preference Procedure (Kemler Nelson et al., 1995) without a familiarization phase. The participants were held on the lap of a caregiver sitting in the center of the test booth, facing the central panel. At the beginning of each trial, the central light blinked until the participant looked in that direction. The green light was then extinguished and the side light above one of the loudspeakers began to flash. When the infant made a turn of at least 30° toward the flashing light, the presentation of the sounds for the given trial was initiated and the red light on that side continued to flash for the entire duration of the trial. If the infant turned away by 30° for less than 2 s and then turned back again, the trial continued but the time spent looking away was not recorded. If the orientation time for a trial was shorter than 1.5 s, the trial was repeated. This minimum orientation time was used to ensure that the participants heard at least one or two words of each list.

Each session began with a training phase made up of two musical trials, allowing the participant to practice the association between headturn and presentation of the stimuli. Each infant listened to all 12 word-lists (six containing ELCs and six containing LLCs) and presentation side varied randomly from trial to trial (three trials on the left side and three trials on the right side for each condition). The order of the lists was pseudorandomized, with no more than two trials in a row of the same condition or on the same side. All infants provided data for all 12 trials.

### Results and discussion

#### Production skills

The mean number of consonants produced in the group amounted to 5.4 (range: 3–8). The majority of the participants produced ELCs (plosives and nasals) while only a minority (seven out of 32 infants) were reported to produce an ELC: the lateral/l/; none was reported to produce fricatives. In summary, differences in production skills between the participants amounted to the number of ELCs in their phonetic inventories. This contradicted our expectations, based on De Boysson-Bardies and Vihman (1991), to find reduced but active production of LLCs at this age. The quantity and types of consonants produced per participant are shown in Table 3.

#### Headturn preference procedure

**FIGURE 1:**
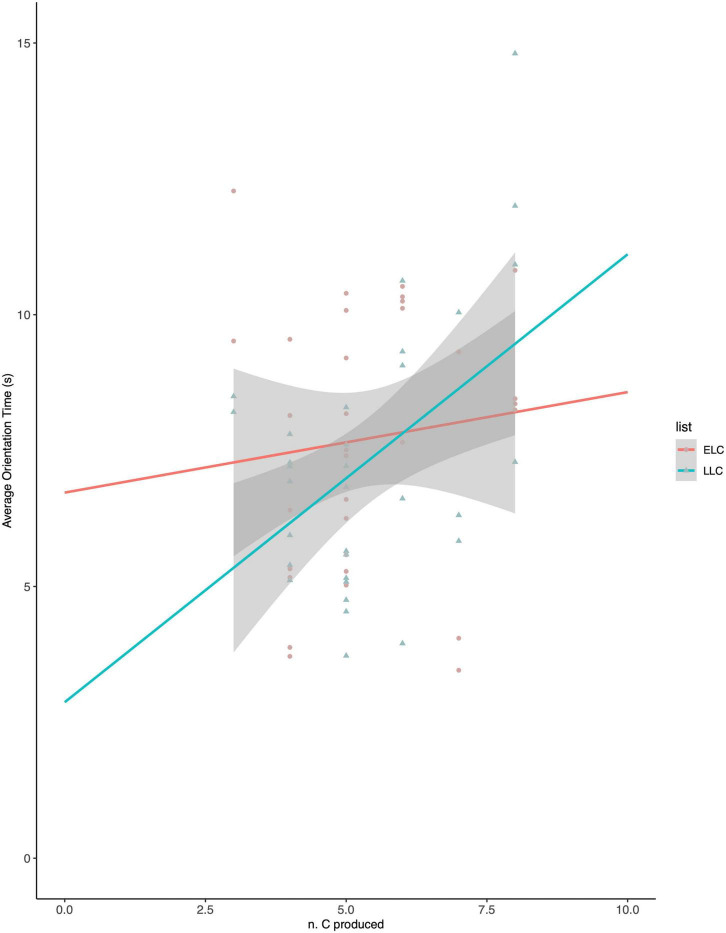
Orientation times to early-learned consonants (ELC) and late-learned consonants (LLC) word–lists at 11 months.

A repeated-measures analysis of covariance (ANCOVA) on orientation times was performed, with type of list (ELC word-forms vs. LLC word-forms) as within-subject variable and production skills (number of produced consonants, centered linear variable) as between-subject variable. The ANCOVA revealed no main effect of either Word-list (*F* = 2.758; *p* = 0.107) or babbling (*F* = 3.834; *p* = 0.060). However, the Word-list x babbling interaction was significant (*F* = 5.497; *p* = 0.026; η^2^ = 0.155), revealing that the effect of babbling on OTs varied for the two word-lists. We performed *post hoc* analyses as simple linear regression targeting the predictive power of babbling on OTs for either the ELC or LLC word-lists. Results show that babbling was a significant predictor of OTs for LLC word-lists (*r^2^* = 0.229; *p* = 0.006) but not for ELC word-lists (*r^2^* = 0.012; *p* = 0.550). In other words, OTs tended to increase with babbling scores (the marginal babbling effect), but the effect of babbling scores on OTs was significantly stronger for LLC vs. ELC word-lists and it was only significant for the LLC word-lists (cf. Figure 1). Accordingly, infants with relatively more advanced babbling oriented more to the LLC word-lists than infants with relatively less advanced babbling.

These results parallel the trends in DePaolis et al. (2011) and Majorano et al. (2014). This notwithstanding, it has to be noted that the relationship between the stimuli used and the babbling patterns observed in the group turned out to be different from our intentions. Our aim was to test the preference found by DePaolis et al. (2011) and Majorano et al. (2014), where “novel” consonants triggered longer orientation times in infants displaying richer babbling patterns. However, these studies identified such effect as long as target consonants were novel but within reach in terms of articulatory skills. Contrary to our expectations, such precondition was seemingly not satisfied in the present experiment. Based on former babbling surveys on French-learning infants, we expected production of a minority of fricatives and liquids to have started at 11 months of age (De Boysson-Bardies and Vihman, 1991). Contrary to our expectation, production of LLCs was very rarely reported in this group of 11-month-olds. Hypothetically, these infants might have been on the verge of producing LLCs that, thus, would have captured their attention following the pattern observed in previous investigations. However, the framework for our predictions being only incompletely fulfilled, we repeated the experiment with a new group of participants aged 14 months. We hypothesized that slightly older infants might have started LCC production and, thus, display a clearer perception-production interaction.

## Experiment 2

### Materials and methods

#### Participants

Thirty-two full-term healthy infants were included in the group (14 females, 18 males; mean age: 14 months, 11 days; range: 13 months, 28 days–14 months, 28 days). All came from monolingual French-speaking homes and had no familial history of speech, language or hearing disorders. Four additional infants were tested but not included due to fussiness. All parents gave written informed consent and completed an information sheet confirming the inclusionary criteria.

#### Production assessment: Parental questionnaire

Same as Experiment 1.

#### Headturn preference procedure

##### Stimuli

The selection of the stimuli followed the same criteria as Experiment 1 but was adapted to fit 14-month-olds. Hence, the test words were not the same (precisely, one of the ELC-words and four of the LLC-words had to be changed to respect our familiarity criteria, cf. Tables 4, 5 for the list of the stimuli used and their acoustic characteristics). Twenty familiar words were selected from the French version of the CDI so as to obtain 10 words exclusively containing ELCs and 10 words exclusively containing LLCs. The words were comprehended, at 14 months, by 46% of French-learning infants on average (range: 22–76%; based on parental reports by Kern, personal communication). This mean comprehension rate is slightly higher than in Experiment 1, due to an overall increase in comprehension rates between the two ages. As in Experiment 1, we asked families whether the infants were able to produce one or more of the test words. Percentage of production of the test words was negligible (two infants out of 32 were reported to say [tête], *head*; 1 infant was reported to say [ga] for [gateau], *cake*).

**TABLE 4:** Test stimuli used in experiment 2: familiar words containing early- vs. late-learned consonants (ELC; LLC).

Familiar word ELC	Phonetic transcription	Syllabic length	English translation	% French-learning infants familiar with the word	Mean familiarity index (scale: 1–3)	Word frequency (Lexique FreqLemFilms2)	Mean diphone frequency
pain	pɛ̃	1	*bread*	67	2	67.58	368
nez	ne	1	*nose*	59	3	75.18	NA
pot	po	1	*jar*	27	1	29.89	794
pied	pje	1	*foot*	67	2	214.08	1586.5
pomme	pɔm	1	*apple*	27	2	42.35	741
tête	tεt	1	*head*	41	3	475.87	1746
cube	kyb	1	*cube*	27	1.2	2.81	566
gâteau	gato	2	*cake*	61	1.8	55.19	863
body	bodi	2	*body*	37	1.5	*NA*	1077
banane	banan	2	*banana*	47	2	11.4	1199

**Familiar word LLC**	**Phonetic transcription**	**Syllabic length**	**English translation**	**% French-learning infants familiar with the word**	**Mean familiarity index (scale: 1-3)**	**Word frequency (Lexique FreqLemFilms2)**	**Mean diphone frequency**

chat	∫ a	1	*cat*	63	3	93	850
lit	li	1	*bed*	76	3	184.27	2843
lait	le	1	*milk*	51	2	59.62	1226
lion	ljɔ̃	1	*lion*	24	1.8	20.86	2652
four	fuR	1	*oven*	22	1	15.44	569.50
vache	va∫	1	*cow*	45	1.5	47.71	1890.50
chaise	∫εz	1	*chair*	55	1.7	40.02	977.50
vélo	velo	2	*bike*	31	2	35.58	861
chausson	∫ osɔ̃	2	*slipper*	57	2	3.5	390
cheval	∫ə val	2	*horse*	39	1.4	129.12	1690
***T*** **-tests (two-sided) ELC vs. LLC words:**				*t*_(18)_ = −0.04; *p* = 0.96	*t*_(18)_ = 0.034; *p* = 0.97	*t*_(18)_ = 0.84; *p* = 0.41	*t*_(18)_ = −1.30; *p* = 0.21

**TABLE 5:** Experiment 2: acoustic characteristics of the stimuli.

	Condition		*t*-value and significance level (two-sided)
	Early-learned C	Late-learned C	
Duration (ms)	540 (SD = 10)	570 (SD = 0.06)	*t*_(18)_ = −0.40; *p* = 0.69
Amplitude (dB)	75 (5.5)	76.9 (2)	*t*_(18)_ = −0.17; *p* = 0.87
F0 mean (Hz)	269.75 (14)	270 (15)	*t*_(18)_ = −0.009; *p* = 0.99
F0 min (Hz)	210.23 (30)	212.6 (32)	*t*_(18)_ = −0.1; *p* = 0.92
F0 max (Hz)	330 (19)	318.7 (21)	*t*_(18)_ = 0.28; *p* = 0.78

As in Experiment 1, the familiarity of the words was verified for each participant. The mean answer was 1.95 (*SD* = 0.63), similar to Experiment 1. Most infants (22) were reported to be familiar with all words, and only 10 infants were reported not to be familiar with all the words (mean unfamiliar words = 3 out of 20).

The order of all items in the lists (including initial items) was pseudo-randomized, verifying that each word was well-distributed within and across lists. All lists lasted 22.07 seconds (interstimulus interval range: 550–630 ms). All subjects provided data for all trials.

### Procedure and apparatus

Same as Experiment 1.

### Results and discussion

#### Production skills

The mean number of consonants produced amounted to 6.2 (range: 3–9). While the variety of consonants produced across participants contained a majority of ELCs (plosives and nasals), 11 out of 32 infants were reported to stably produce at least one LLC, specifically, /l/and/or one fricative (/∫/,/s/,/ᴚ/or/z/). Thus, production skills at 14 months fitted better than at 11 months with the schema that we had planned for our stimuli: at the group level, ELC production was mastered; LLC production was at its beginning but more advanced and varied in comparison with the 11-month-old group (in which no participant produced any fricative consonant). The quantity and type of consonant produced are shown in Table 6.

**TABLE 6:** Experiment 2: quantity and type of consonant produced (parental questionnaires. 14-month-olds).

Age (mos.days)	Tot. C produced	Babbling inventory
14.9	3	t d m
14.5	3	p b m
14.11	3	b d m
14.28	4	p b m n
14.13	4	p t d m
14.15	4	p b m l
14.5	5	p b k m l
14.6	5	p b t d m
14.28	5	p t k m n
14.6	5	p b d k g
14.10	5	p d k m n
14.25	6	p b t d m n
14	6	p b t d g m
14.18	6	p b d k g m
14.15	6	p t b d k m
14.7	6	b t d g m n
14.14	6	p b t k m n
13.27	6	p b t d g m
14	7	p b t d m n l
14.16	7	p b t d k g m
14.24	7	p b t d k g m
14.20	7	p b t d m n ∫
14.12	7	p b t k g m n
14.15	7	p b t k g m n
14.2	8	p b t d g m n s
14.3	8	p b t d k m n l
14.13	8	b t d k g m n l
13.28	8	p b t d g k m n
14.9	9	p b t d k g m n l
14.14	9	p b t d k m n l ᴚ
14.8	9	p b t d k g m n z
14.18	9	p b t d k g m n l

Although this is a cross-sectional study and, thus, it does not capture developmental trajectories, note that infants falling at the lower end (i.e., below the median value) of production skills at 14 months still produced fewer speech sounds than infants falling at the upper end of the range at 11 months (4.9 vs. 6.9 respectively): across the two age-groups, speech sensorimotor development appears age-independent. This highlights large interindividual variability in early babbling development, and further studies would be needed to identify the causes of such variability, as this is beyond the scope of the present study.

#### Headturn preference procedure

As in Experiment 1, a repeated-measures analysis of covariance (ANCOVA) on orientation times was performed, with type of list (ELC word-forms vs. LLC word-forms) as within-subject variable and production skills (n. of produced consonants, centered linear variable) as between-subject variable. The ANCOVA revealed no main effect of either Word-list (*F* = 0.867; *p* = 0.359) or babbling (*F* = 1.823; *p* = 0.187). The Word-list x babbling interaction was significant (*F* = 4.664; *p* = 0.039; η^2^ = 0.135), revealing that the effect of babbling on OTs varied for ELC vs. LLC word-lists. We thus performed *post hoc* analyses as simple linear regression targeting the predictive power of babbling on OTs for either ELC or LLC word-lists, showing that babbling was a significant predictor of OTs for LLC word-lists (*r^2^* = 0.135; *p* = 0.038) but not for ELC word-lists (*r^2^* = 0.007; *p* = 0.649).

**FIGURE 2:**
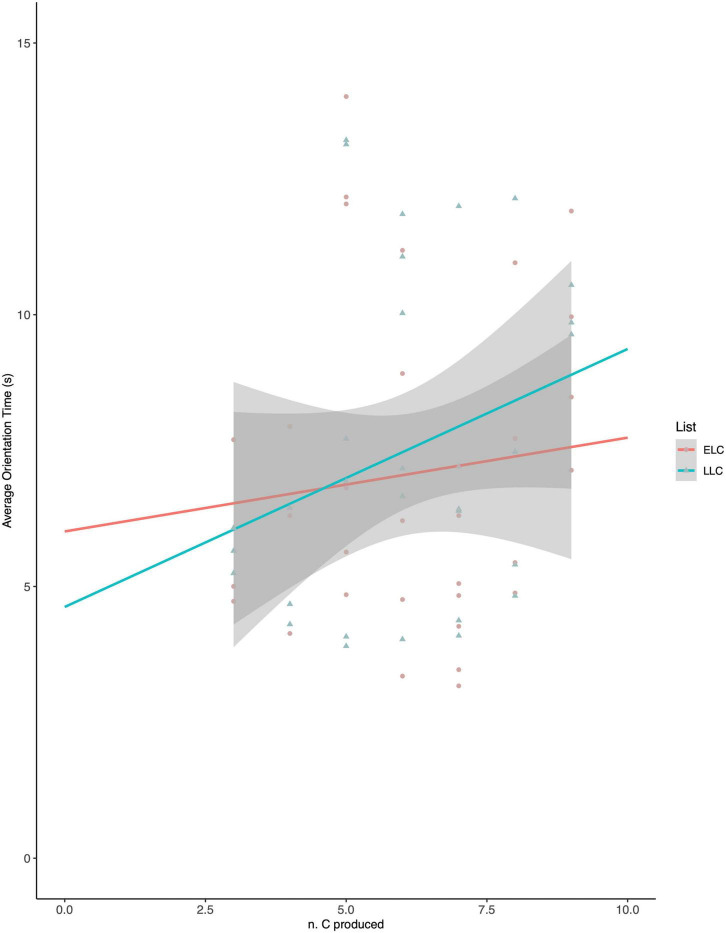
Orientation times to early-learned consonants (ELC) and late-learned consonants (LLC) word–lists at 14 months.

Again, as seen in Figure 2, infants with more advanced babbling looked more to the target-words for LLC word-lists.

As in Experiment 1, babbling abilities modulated orientation times for the LCC word-lists, with longer orientation times in more advanced babblers. Importantly, here, our stimuli fitted more accurately the precondition outlined by DePaolis et al. (2011) that “novel” consonants should be within reach of the participants’ articulatory abilities. Indeed, at the group level, 14-month-olds mastered ELCs, and participants with relatively more advanced babbling skills had begun producing LLCs, including fricatives. Thus, the results obtained in this experiment replicate and confirm the pattern observed at 11 months. Overall, this study extends the previously observed listening preference for non-mastered (yet producible) consonants in infants with more advanced babbling to familiar word-form recognition. Importantly, this effect is found in a procedure that does not require word-form encoding.

## General discussion

Former investigations have shown differential processing of newly encoded word-forms as a function of production abilities in infancy (DePaolis et al., 2011; Majorano et al., 2014). Here, we presented infants with lists of known word-forms made up of Early-Learned consonants (ELCs, that is, plosive or nasal consonants) and lists of known words made up of Late-Learned consonants (LLCs, that is, fricatives or the lateral/l/), and measured their attention to these two types of lists according to their babbling skills. We show that similar effects of production abilities hold for the processing of real familiar word-forms learned from the daily environment prior to coming to the lab.

Specifically, at both 11 and 14 months of age, we found that attention to LLC consonants was modulated by babbling abilities. However, at 11 months of age (Experiment 1), the preconditions of our experimental design were not entirely fulfilled. Specifically, following previous literature, we wanted to contrast well-mastered consonants (ELCs, in our stimuli) with consonants that were not yet mastered, but were within reach in terms of articulatory abilities (LLCs). However, LLC production was very rarely reported in this group, and, in particular, fricative production was entirely absent. To explain the effect of babbling on perception found at that age based on previous literature (e.g., DePaolis et al., 2011), we needed to assume that the 11-month-olds tested were on the point to produce fricatives, thus that fricatives could be considered as consonants rarely produced but within reach for the participants’ articulatory abilities. However, this point remained speculative. To clarify this issue, we tested an older group of 14-month-olds (Experiment 2), for whom LLC production was more productive and varied, and included fricatives. Results replicated the findings of Experiment 1.

Overall, Experiment 1 and 2 parallel and extend the results of DePaolis et al. (2011) and Majorano et al. (2014) demonstrating that the perception/production effect is at play in word-form recognition when the encoding phase is excluded from the experimental procedure.

The encoding/recognition distinction given by our results is important, in that it shows that infants store speech-related sensorimotor information in long-term word-form representations, and access this knowledge during word-form processing. Specifically, not only do they use sensorimotor information during phonological encoding [as shown in seminal studies by DePaolis et al. (2011) and Majorano et al. (2014)], but they appear to also store sensorimotor information in long-term representations and access such information during recognition. Our results support views according to which, when frequent phonological chunks start to be retained in long-term memory, the (multisensory) phonological representation of such items include sensorimotor information extracted from experience with speech sound articulation (among which, the Articulatory Filter Hypothesis). Indeed, the perception/production effect found in this study was observed at 11 months, the youngest age at which untrained familiar word-form recognition has been reported (Vihman, 2022). This shows that sensorimotor knowledge of speech sounds is stored in word-form representations since their onset.

To sum up, distinguishing recognition from encoding, in this context, has theoretical relevance for the perception/production relationship. In fact, it allows to explore whether sensorimotor information modulating consonant processing is part of what is retained in linguistic representations (and, thus, retrieved during recognition) or is processed when learning a new item (during encoding) but not necessarily retained in long-term memory. By showing that sensorimotor information is used for familiar word-form recognition, the present study shows that sensorimotor information is part of word-form memory traces. This is relevant to previous work supporting multisensoriality in early lexical representations but not directly exploring the role of the sensorimotor component (Havy et al., 2017). Complementarily, future investigations might try to isolate further the effect of speech-related sensorimotor information on word-form encoding *vs.* recognition, in order to evaluate the relative contribution of sensorimotor information in the two processes. This might be done through neurofunctional procedures exploring, for example, the different ERP components elicited in word learning, in more *vs*. less-advanced producers and for words containing produced *vs*. non-produced consonants [e.g., by mixing procedures such as those in Mills et al. (2005); Friedrich and Friederici (2008), and Von Holzen et al. (2018)]. In this connection, some details regarding the difficulty to distinguish recognition from encoding with behavioral studies are also worth discussing. First, it is important to underline that the experimental distinction between word-form encoding and recognition can be graded in subjects who are in the process of developing a lexicon, for whom different word-forms can have stronger or weaker memory traces. The consequence for our looking-time study is that, while the recorded orientation times should always reflect recognition (as we verified phonological familiarity of the items presented), they might, in some cases and to some extent, also capture a form of refreshing of the encoding phase (depending on the degree of familiarity of specific stimuli for a specific infant). Relatedly, it should also be considered that encoding is a prerequisite to recognition, so that goodness of encoding can affect efficiency in recognition (the better the former, the better the latter). Overall, the experimental distinction between these two processes is not without challenge and future investigations are needed, contrasting, for example, the presentation of familiar and unfamiliar stimuli containing either early- or late-learned consonants.

Our study is also relevant to the results obtained by Majorano et al. (2019) and Laing and Bergelson (2020). The authors formerly addressed the impact of production practice on long-term word-form retention: Majorano et al. with a word-learning task; Laing and Bergelson analyzing the similarity between consonants produced in babbling and the name of the object on which infant’s attention was focused while babbling. Both studies suggested a perception/production relationship in word retrieval, but failed to report strong and consistent effects of the participants’ specific babbling patterns. Our results show consistent and specific perception/production links, starting from 11 months, for long-term representations at the word-form (phonological) level. This encourages further research clarifying the reasons why such production effects have less readily emerged in investigations where the level of meaning was also at play.

With this investigation, we extend the novelty effect previously found for newly encoded pseudowords (DePaolis et al., 2011; Majorano et al., 2014) to familiar word-form recognition. At both 11 and 14 months of age, infants with more advanced babbling oriented more to familiar word-forms embedding ‘novel’ consonants than infants with less advanced babbling. This pattern of preference establishes an effect of speech-related sensorimotor knowledge on long-term speech sound representations.

## Data availability statement

The raw data supporting the conclusions of this article will be made available by the authors, without undue reservation.

## Ethics statement

The studies involving human participants were reviewed and approved by Conseil d’évaluation éthique pour les recherches en santé (CERES) Paris Descartes. Written informed consent to participate in this study was provided by the participants’ legal guardian/next of kin.

## Author contributions

IL: experimental design, testing, analyses, and manuscript writing. TN: supervision of experimental design and manuscript writing. Both authors contributed to the article and approved the submitted version.
